# The effectiveness and cost-effectiveness of plant sterol or stanol-enriched functional foods as a primary prevention strategy for people with cardiovascular disease risk in England: a modeling study

**DOI:** 10.1007/s10198-017-0934-2

**Published:** 2017-11-06

**Authors:** Wei Yang, Heather Gage, Daniel Jackson, Monique Raats

**Affiliations:** 1 Department of Global Health and Social Medicine, King’s College London, London, WC2R 2LS UK; 2 School of Economics, University of Surrey, Guildford, Surrey, GU2 7XH UK; 3 School of Psychology, Faculty of Health and Medical Sciences, Food, Consumer Behaviour and Health Research Centre, University of Surrey, Guildford, Surrey, GU2 7XH UK

**Keywords:** Plant sterols, Cardiovascular disease risk, England, Cost-effectiveness analysis, I19

## Abstract

This study appraises the effectiveness and cost-effectiveness of consumption of plant sterol-enriched margarine-type spreads for the prevention of cardiovascular disease (CVD) in people with hypercholesterolemia in England, compared to a normal diet. A nested Markov model was employed using the perspective of the British National Health Service (NHS). Effectiveness outcomes were the 10-year CVD risk of individuals with mild (4–6 mmol/l) and high (above 6 mmol/l) cholesterol by gender and age groups (45–54, 55–64, 65–74, 75–85 years); CVD events avoided and QALY gains over 20 years. This study found that daily consumption of enriched spread reduces CVD risks more for men and older age groups. Assuming 50% compliance, 69 CVD events per 10,000 men and 40 CVD events per 10,000 women would be saved over 20 years. If the NHS pays the excess cost of enriched spreads, for the high-cholesterol group, the probability of enriched spreads being cost-effective is 100% for men aged over 64 years and women over 74, at £20,000/QALY threshold. Probabilities of cost-effectiveness are lower at younger ages, with mildly elevated cholesterol and over a 10-year time horizon. If consumers bear the full cost of enriched spreads, NHS savings arise from reduced CVD events.

## Introduction

Raised total or low-density-lipoprotein cholesterol (LDL-c) is a major risk factor predisposing an individual to cardiovascular disease (CVD), which can be modified by various prevention programs, such as changes in diet. Plant sterols and stanols (a saturated subgroup of sterols), hereafter referred to collectively as plant sterols, are plant equivalents of cholesterol with a very similar molecular structure [1]. They are found naturally in fruit, vegetables, nuts, seeds, grains, and legumes and prevent the absorption of cholesterol into the bloodstream, but are unlikely to be consumed in sufficient quantities to reduce cholesterol levels [2–4]. Research has shown that adding plant sterols into the daily diet can substantially enhance the cholesterol-lowering effects of diet change [2–5]. Functional foods enriched with plant sterols, including margarine-type spreads, mayonnaise and salad dressing, and dairy products (milk, yogurt, cheese), have been shown to achieve a beneficial effect on the serum lipid profile of the consumer [6].

Although the effectiveness of plant sterols in reducing LDL-c has been verified in a number of studies [2, 3, 7–13], evidence on whether use of plant sterols is a cost-effective preventive strategy for reducing CVD risks is limited. To date, only four modeling studies have been identified but their findings may not be applicable to the United Kingdom (UK) context [14–17]. Three of these studies based their CVD risk estimations on the Framingham equation, which has been shown to overestimate CVD risks for the UK population and is no longer recommended by the National Institute of Health and Clinical Excellence (NICE) [18, 19]. Moreover, health states in some studies are simplified for the convenience of the analysis [14, 16]. Also, it is instructive to look at the cost-effectiveness separately for different age and gender groups and at different compliance levels.

The study reported in this paper used a nested Markov model to assess the effectiveness and cost-effectiveness of plant sterol-enriched functional foods for the prevention of CVD disease in the English population with hypercholesterolemia, when compared to a normal diet (no plant sterol-enriched functional foods). The analysis takes the perspective of the British National Health Service (NHS), and considers costs borne by consumers for the purchase of functional foods. Health outcomes are represented by CVD events, mortality, and quality-adjusted life years (QALYs). Cost-effectiveness is defined by the NICE threshold of between £20,000 and £30,000 per QALY gained [20].

## Methods

### Model structure

A decision analytical model was used to synthesize epidemiological, clinical, and economic data to appraise the effectiveness and cost-effectiveness of plant sterol-enriched functional foods in the prevention of CVD in England. A nested Markov model structure, which allows the occurrence of both primary and secondary CVD events, was derived from a model used previously for a health technology assessment of the impact of statins [21]. All individuals start in the event-free (EF) health state. During each annual cycle of the model, individuals (depending on their risk) either remain EF or have a primary event and enter one of the event health states: stable angina, unstable angina, non-fatal myocardial infarction (MI), transient ischemic attack (TIA), non-fatal stroke, or death (either due to CVD or other causes). In each subsequent cycle, individuals in a non-fatal CVD event health state may move to a secondary event state, as shown in Table 1.

**Table 1:** Model structure

Primary events		Secondary events	
From	To	From	To
Event-free	Event-free		
	Stable angina	Stable angina	Stable angina
			Unstable angina
			Non-fatal MI
			Death
	Unstable angina	Unstable angina	Post-unstable angina
			Non-fatal MI
			Death
	Non-fatal MI	Non-fatal MI	Post non-fatal MI
			Non-fatal MI
			Non-fatal stroke
			Death
	TIA	TIA	Post-TIA
			Non-fatal MI
			Non-fatal stroke
			Death
	Non-fatal stroke	Non-fatal stroke	Post non-fatal stroke
			Non-fatal MI
			Non-fatal stroke
			Death
	Death		

### Population

The analysis focused on individuals aged 45 and above with baseline total cholesterol level ≥ 4 mmol/l [22]. Based on evidence in a recent meta-analysis, it was assumed that plant sterols are a primary prevention strategy and effective only for people in the EF state [4]. Hence, those with a history of CVD were excluded. The baseline cohort was drawn from the Health Survey for England (HSE) 2011, an annual survey conducted by the Health and Social Care Information Centre that uses random samples of the population living in private households to gather information about the nation’s health. In particular, the HSE 2011 focused on CVD and gathered information on an individual’s risk factors, including cholesterol level, CVD history, and other relevant health and demographic variables. Data from the HSE 2011 have been used to model CVD risks in various health economic studies [23, 24].

Two clinical scenarios were considered: individuals with cholesterol levels between 4 and 6 mmol/l (mildly elevated cholesterol population) and those with cholesterol levels above 6 mmol/l (high-cholesterol population) [22]. For each scenario, the population was modeled separately by gender and age group (45–54, 55–64, 65–74, 75–85 years). The age range was determined by the QRISK2 function (www.qrisk.org), which was used to predict CVD risks, and which only extends to 85 years.

### Estimation of CVD risk and other-cause mortality

The primary estimation of CVD risk for the study population was based on the QRISK2 equation (QRISK2-2014). Recommended by NICE, QRISK2 is a new CVD risk prediction tool which provides a 10-year CVD risk estimation for the UK population (www.qrisk.org) [25]. It is a validated tool and has been used in various clinical studies [18, 19]. QRISK2 predicts CVD risks based on a wide range of risk factors including age, systolic blood pressure, smoking status, ethnicities, ratio of total serum cholesterol to high-density lipoprotein, body mass index, family history of coronary heart disease in first-degree relative, body mass index (BMI), Townsend deprivation score, treated hypertension, and diagnosis of rheumatoid arthritis, atrial fibrillation, type 2 diabetes, and chronic renal disease.

The 10-year individual risk prediction provided by QRISK2 was converted to a 1-year risk for each gender and age subgroups. The conversion is based on published and validated algorithms [16, 26]. The QRISK2 tool indicates the probability of a CVD event occurring, but not the type of event. The distribution of types of events within groups, and the transition probabilities to model the number of people moving from any particular health state to another over subsequent cycles, were based on the probabilities in a study conducted by Ward et al. [21]. The percentage of people dying from non-CVD causes was accounted for using age- and gender-specific mortality rates derived from Office of National Statistics data for 2015 [27]. Annual risks of non-CVD deaths were estimated from the causes of death register by subtracting the fraction of deaths due to CVD causes from the total mortality.

### Clinical effectiveness of plant sterols on cholesterol lowering

The dose–response relationship for the cholesterol-lowering effect of plant sterols has been explored in a number of meta-analyses [2, 7, 9]. It is suggested that the LDL-c-lowering effect starts at intakes of 2–3 g/day with little additional benefit at higher intakes [1, 7]. It is also suggested that incorporating higher amounts of plant sterols into foods is technically unrealistic [28, 29]. Several health authorities include 3 g/day plant sterols from enriched foods as part of their diet and lifestyle guidelines in the management of hypercholesterolemia [30, 31]. Therefore, this study used 3 g/day as the dose value for plant sterols in the model.

A literature search was undertaken to identify the clinical effectiveness of plant sterols. A scientific opinion by the European Food Safety Authority (EFSA) suggested that an intake of plant sterols of 3 g/day (2.6–3.4 g) reduced the LDL-c levels effectively by 11.2–11.4% (95% CI 9.8–13.0), and that the minimum duration required to achieve the maximum effect is 2–3 weeks [4]. The conclusions of the effect size were consistent with other findings [2, 7]. The most recent clinical evidence (up to 2014) is provided by the meta-analysis of 129 studies by Ras et al. [9] concluding that intakes of approximately 3 g/day (plant sterols) led to an average LDL-cholesterol-lowering effect of 12%, and this was used as the basis for the modeling. In the absence of evidence for the time course of intervention effects, it was assumed that the protective effect of plant sterols continues, providing minimum intakes are maintained.

### Impact of plant sterols on relative risk

The 10-year CVD risk was re-calculated for each individual in the study population using the QRISK2 assessment tool, and grouped by gender and age (as described above), assuming a 12% reduction to total cholesterol or LDL-c level caused by consuming 3 g/day of plant sterols. Relative risk (RR) was calculated as the ratio of the probability of a CVD event occurring with consumption of plant sterols to the probability of the event occurring in the non-exposed (normal diet) condition, for each gender and age group. Relative risks were used in the simulation, with standard errors of RRs taken into account to reduce parameter uncertainties. In this way, the difference in number of events between the functional food and normal diet conditions, and specifically the number of events avoided by sterol consumption, were identified.

### Costs of health states and functional foods

One output of the model was annual numbers of individuals in different health states to which unit costs of treatment were applied. The cost of an event was included in the year in which it occurred, and a maintaining cost was applied in subsequent years. Costs of health states were largely obtained using 2014 NHS reference costs [32]. Where cost information was not available, costs from a published health technology evaluation of statins were used [21]. Information on the cost of health states can be found in Appendix 1.

Commonly available foods enriched with sterols include margarine-type spreads, yogurt, and milk. The analysis was based on spread, as this is the product that enables the required dose of plant sterols (3 g per day) to be consumed at the least cost. Margarine-type spreads are common items in the diet of the British population with median consumption in adults around 40 g per day [33], which is sufficient to ensure an intake of 3 g of sterols (Appendix 2). The costs of products were obtained from the websites of three national supermarket chains in the UK in April 2015, with the supermarket’s own brand used for the non-sterol-enriched spread. Unit costs were the same across retailers for each product. Calculation of food costs can be found in Appendix 2.

### Quality-adjusted life years (QALYs)

Utility estimates for health states were derived from various sources following a review of the literature that focused on UK-based studies and use of the preference-based utility instrument, the EQ-5D, which is the recommended instrument for measuring QALYs [34–36]. Health utilities used in the analysis can be found in Appendix 1. These utility values were applied to annual health states for individuals, and a mean value was calculated.

### Main analysis

The baseline population was described using summary statistics. CVD risks and RRs for the normal diet group and plant sterol condition were compared. A cost-effectiveness analysis was then conducted from the NHS perspective. The price of spread enriched with plant sterols is higher than that of non-enriched spread and this may discourage purchases. Therefore, in the base case, it was assumed that the NHS would subsidize the cost difference between the supermarket’s own brand of non-enriched spread (£54.10 pa) and the manufacturer-brand plant sterol spread (£111.04 pa), i.e., £56.94 pa, for each of the 20 years of the modeling (Appendix 1). Two compliance rates—10 and 50%, based around pessimistic and ideal levels identified in a Canadian study—were explored [17]. The modeling was conducted over 20 years until the average age of the baseline cohort reached the life expectancy of the UK population (85 years). In line with NICE recommendations for health technology assessments, a discount rate of 3.5% was used for both costs and utilities [37].

The number of events avoided by consuming plant sterols over the modeling period was calculated by a deterministic model. Incremental costs, incremental QALYs, and the incremental cost-effectiveness ratios (ICERs) were then calculated for each gender and age group. Incremental costs from the plant sterol diet, compared to the normal diet, were calculated as the cost of the NHS subsidy for the spread over the 20-year period less any treatment cost savings from reduced CVD events. Incremental QALYs are the difference in QALYs between the plant sterol group and the normal diet group. ICERs show the cost per QALY gained when a diet enriched with plant sterols is followed, rather than a diet without the functional food. Uncertainty around point estimates in the cost-effectiveness analysis was examined using probabilistic sensitivity analysis (PSA), and cost-effectiveness acceptability curves (CEAC) were plotted. For each age and gender group, a second-order Monte Carlo simulation using the probabilistic parameters based on 5000 replications was carried out [26].

### Sensitivity analysis

Four sets of sensitivity analyses were conducted. First, a one-way sensitivity analysis related to the assumed clinical efficacy of plant sterols was conducted using the estimated upper (13.3%) and lower (10.7%) limits of 95% confidence intervals around the average LDL reduction level of 12% in the paper [9]. Second, it was assumed that the NHS pays the full food costs, and the compliance level was 50%. Third, it was assumed that individuals were responsible for the full costs of the plant sterol-enriched spread (no NHS subsidy), and for this model a very pessimistic scenario of 5% compliance was used [17]. Lastly, to align with the 10-year individual risk protection provided by QRISK2, ICERs were recalculated for the base case (NHS pays the excess cost of sterol enriched margarine) over a 10-year time horizon, at 50% compliance.

All analyses were carried out using STATA13 and Microsoft Excel 2013. Half cycle correction was used for costs and utilities. Detailed information of model parameters and distribution is shown in Appendix 3.

## Results

### Baseline description of study population

There were 1598 people with mildly elevated cholesterol (4–6 mmol/l) and 640 with high cholesterol (above 6 mmol/l) in the HSE 2011. The key risk factors are summarized in Table 2. The mean BMI, systolic blood pressure, and total cholesterol/HDL cholesterol ratio was higher in the high-cholesterol group, which also contained a larger proportion of women than the mild cholesterol group. Applying QRISK2, the average 10-year CVD risk for the mild-cholesterol group is 12.27, and 12.85% for the high-cholesterol group.

**Table 2:** Baseline characteristics of modeled population by cholesterol level

	Mild-cholesterol population (*N* = 1598)		High-cholesterol population (*N* = 640)	
	Mean	SD	Mean	SD
Age	59.40	10.42	60.46	9.91
Proportion of male	0.41	0.49	0.35	0.48
BMI	27.61	4.95	28.04	4.90
Systolic blood pressure (mmHg)	129.73	17.41	132.10	17.41
Total cholesterol (mmol/l)/HDL ratio (mmol/l)	3.98	1.38	4.51	1.53
10-year CVD risk	12.27	11.87	12.85	10.78

### Risks and events avoided

**Fig. 1:**
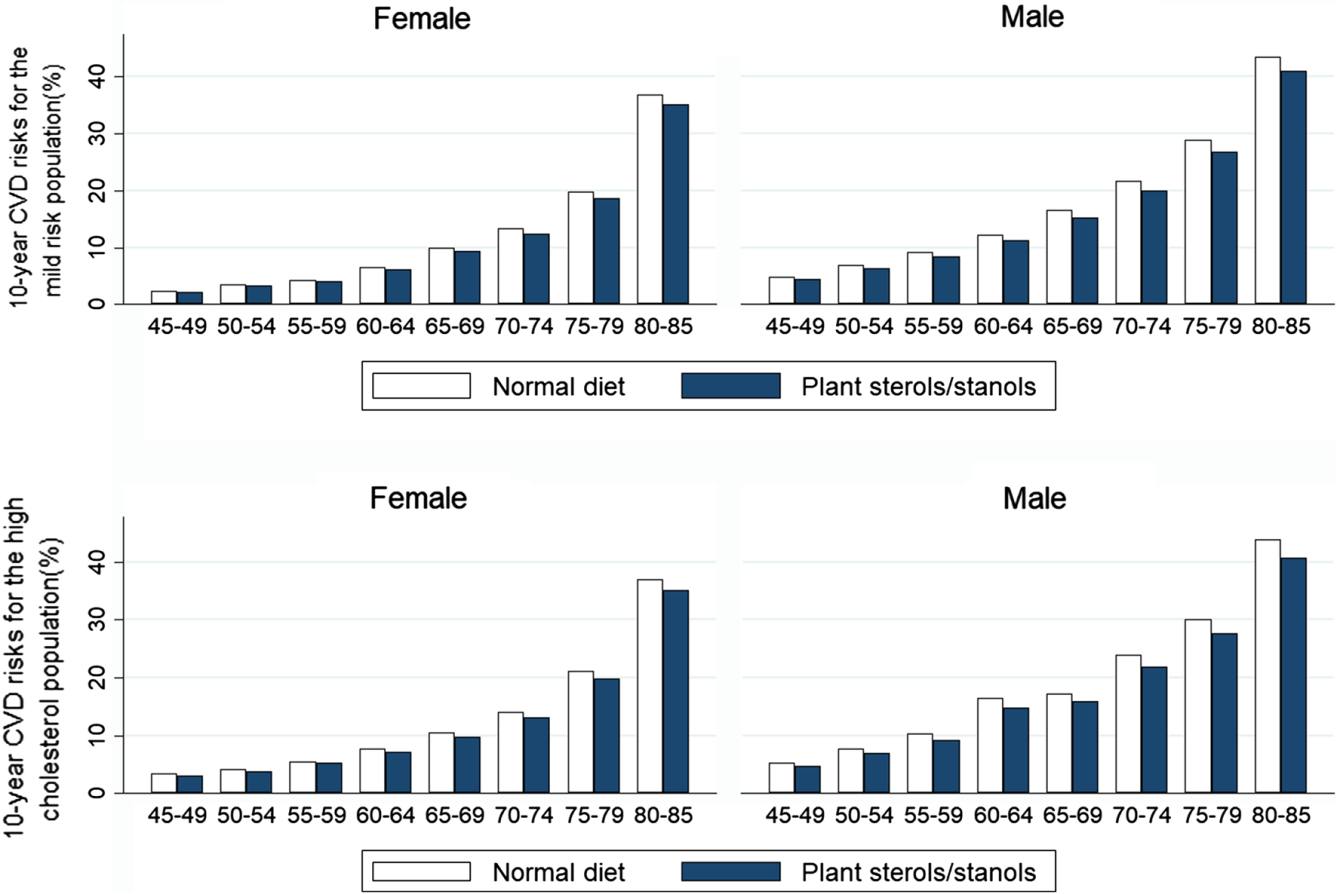
Ten-year CVD risk for the mild- and high-cholesterol groups for the normal diet and plant sterol groups by age and gender

For both cholesterol groups, the 10-year CVD risks increase with age. The plant sterol group is associated with lower 10-year CVD risks than the normal diet group at all ages (Fig. 1). Regarding relative risks, plant sterol-enriched functional foods reduce CVD risk more in men than women, and in older age groups, compared to the younger ones (Table 3).

**Table 3:** RR by risk groups by age and gender

	Mild-cholesterol population				High-cholesterol population			
	Male		Female		Male		Female	
	Mean (%)	95% CI	Mean	95% CI	Mean (%)	95% CI	Mean	95% CI
45–54	91.29	0.909, 0.917	94.00	0.938, 0.942	89.99	0.893, 0.907	92.81	0.923, 0.933
55–64	91.65	0.913, 0.920	93.93	0.937, 0.942	89.90	0.892, 0.906	93.30	0.930, 0.936
65–74	91.90	0.915, 0.923	93.62	0.934, 0.939	91.18	0.906, 0.918	93.03	0.927, 0.934
75–84	92.07	0.916, 0.926	94.00	0.937, 0.943	91.00	0.900, 0.920	93.42	0.930, 0.938

Using deterministic parameters, a diet including the recommended levels of plant sterols avoids 69 CVD events (59 non-fatal and ten fatal CVD events) per 10,000 men and 40 (33 non-fatal and seven fatal) per 10,000 women, aged 45–85, at 50% compliance level, and 14 CVD events per 10,000 men and eight CVD events per 10,000 women at 10% compliance level.

### Cost-effectiveness—base case

The age- and gender-specific QALYs, costs, and ICERs (costs per QALY gained from sterol-enriched spread, compared to normal spread) for different cholesterol populations over 20 years at 10 and 50% compliance levels, assuming the NHS pays the excess cost of the sterol-enriched product are shown in Table 4. The cost to the NHS of subsidizing sterol-enriched spread is lower in men because more CVD events are avoided than in women. Accordingly QALY gains, which rise with compliance level and age, are also higher for men.

**Table 4:** Age- and gender-specific ICERs (£/QALY) over 20 years for mild- and high-cholesterol groups at 10 and 50% compliance levels: main analysis in which NHS pays excess cost of sterol-enriched spread

	Mild-cholesterol population								High-cholesterol population					
	Control group QALY	Control group cost	10% compliance			50% compliance			10% compliance			50% compliance		
			Incremental QALY	Incremental cost (£)	ICER: £/QALY	Incremental QALY	Incremental cost (£)	ICER: £/QALY	Incremental QALY	Incremental cost (£)	ICER: £/QALY	Incremental QALY	Incremental cost (£)	ICER: £/QALY
Male														
45–54	12.017	351.79	0.001	71.81	69,091.26	0.005	359.76	69,121.05	0.002	71.38	43,773.69	0.008	357.74	43,790.41
55–64	10.880	586.11	0.002	64.32	37,683.40	0.009	322.74	37,715.67	0.003	63.42	*22,793.76*	0.014	318.45	*22,812.52*
65–74	9.068	860.13	0.003	52.52	**19,714.31**	0.013	264.04	**19,740.48**	0.004	52.03	**13,589.23**	0.019	261.70	**13,604.39**
75–84	6.506	880.86	0.002	37.19	**16,711.10**	0.011	187.35	**16,768.41**	0.003	36.47	**11,923.24**	0.015	183.91	**11,967.03**
Female														
45–54	12.142	185.60	0.000	76.06	223,033.15	0.002	380.54	223,064.18	0.001	75.84	135,436.10	0.003	379.53	135,453.23
55–64	11.134	362.13	0.001	70.78	104,760.51	0.003	354.37	104,788.67	0.001	70.57	69,283.84	0.005	353.37	69,298.86
65–74	9.448	663.35	0.002	59.64	33,269.93	0.009	299.09	33,287.32	0.003	59.31	*22,526.23*	0.013	297.53	*22,534.68*
75–84	6.779	753.95	0.002	42.97	*22,811.15*	0.009	215.86	*22,855.54*	0.003	42.62	**16,659.74**	0.013	214.20	**16,692.58**

The* numbers in italics* are plant sterol-enriched spread cost-effective at NICE threshold below £30,000 per QALY gained

The* numbers in bold* are plant sterol-enriched spread cost-effective at NICE threshold below £20,000 per QALY gained

Incremental QALYs show QALY gained from sterol-enriched spread compared to no sterol-enriched spread

Incremental costs show the cost to the NHS of sterol-enriched spread, less treatment cost savings from reduced CVD events

The ICERs (costs per QALY gained) are higher for mildly elevated cholesterol than for the high-cholesterol group. Hence, subsidizing sterol-enriched spread is more cost-effective at higher cholesterol levels. In both the 10 and 50% compliance models, the cost per QALY gained is below the £20,000 threshold for men over 64 years and women over 74 years with high cholesterol; it is below the £30,000 threshold for men over 54 and women over 64 (Table 4).

**Fig. 2:**
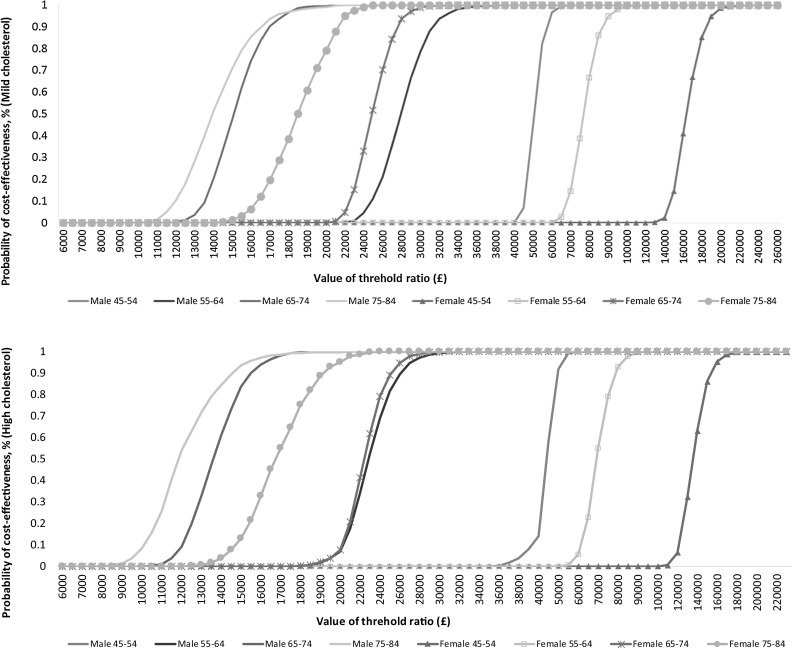
Cost-effectiveness acceptability curves at 10% compliance level (mild- and high-cholesterol population): main analysis in which NHS pays excess cost of sterol-enriched spread

**Fig. 3:**
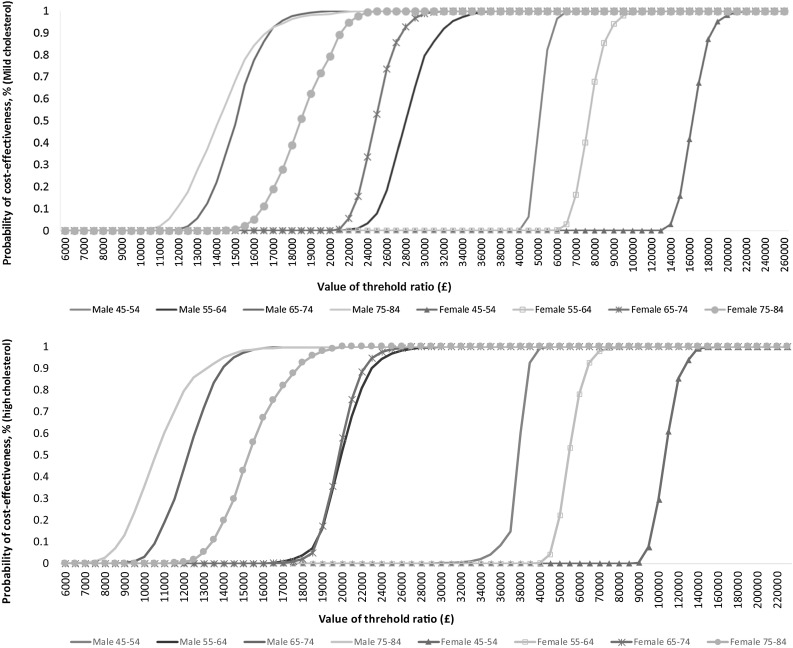
Cost-effectiveness acceptability curves at 50% compliance level (mild- and high-cholesterol population): main analysis in which NHS pays excess cost of sterol-enriched spread

The cost-effectiveness acceptability curves (CEACs) for different age and gender groups at 10 and 50% compliance levels for the high- and mild-cholesterol groups are shown in Figs. 2 and 3. For the high-cholesterol group, when the NHS pays the excess cost, the enriched spread is likely to be cost-effective for men over the age of 64, and women over 74, at the £20,000 threshold, and for men over 54 and women over 64 at the £30,000 threshold, at both the 10 and 50% compliance levels. For the mild-cholesterol group, the probability that plant sterol-enriched spread is cost-effective for any age/gender group, compliance level or threshold is lower than for the high-cholesterol group.

### Sensitivity analysis

Using the upper limit (13.3%) of the 95% confidence interval instead of the assumed mean LDL reduction (12%) from use of plant sterols, with 50% compliance, and the NHS covering the excess cost, did not affect the groups for which enriched spread was cost-effective. However, with the lower limit of the 95% confidence interval, the likelihood of plant sterol-enriched spread being cost-effective is slightly increased for the mild-cholesterol group (Appendix 4).

When the assumption is adopted that the NHS is responsible for the full costs of providing sterol-enriched spread, and the compliance rate is 50%, the plant sterol-enriched diet is only cost-effective for men over 64 with high cholesterol and between 75 and 85 with mild cholesterol, and if the cost-effectiveness threshold is set at £30,000 per QALY gained. It is not cost-effective for women in any age group, or for either men or women at the lower threshold of £20,000 per QALY gained (Appendix 4).

If individuals are assumed to be responsible for the full cost of the sterol-enriched spread, the NHS realizes savings from reduced treatment costs due to fewer CVD events whilst incurring no charges for the products, even at a very pessimistic compliance rate of 5% (Appendix 4).

With a 10-year time horizon (instead of 20 years), at 50% compliance, sterol-enriched margarine becomes less cost-effective for all groups (Appendix 4).

## Discussion

### Summary of main findings

This study is among the first to model CVD outcomes from consumption of plant sterol-enriched foods, and appraise cost-effectiveness within the British NHS for an adult population with hypercholesterolemia. Multiple scenarios were considered involving varied cost-sharing arrangements between the consumer and the NHS (to affect consumption), and different assumptions about levels of compliance. Of several sterol-enriched foods available, the analysis was based on margarine-type spreads because these are commonly consumed, and an efficacious ‘dose’ of sterols is achievable within average daily consumption levels. Moreover, enriched spread is the cheapest means of providing the required intake.

Under the assumption that the difference in cost to the consumer between sterol-enriched and normal spread is subsidized, then the sterol-enriched spread is likely to be cost-effective for men with hypercholesterolemia over the age of 64, and for women with hypercholesterolemia over 74 years with the compliance level of 10 and 50% and the cost-effectiveness threshold of £30,000 per QALY gained. At the lower threshold of £20,000 per QALY gained, the subsidy is less likely to be cost-effective at lower age groups. Reducing the time horizon for the modeling diminishes the likelihood of sterol-enriched spread being cost-effective. Shifting the cost burden of the product to consumers increases the likelihood of cost-effectiveness. Ultimately, if consumers in the target groups bear the full cost, the NHS will maximally benefit from reduced CVD treatment costs. The other more costly sterol-enriched products are likely to be less cost-effective for the NHS.

### Comparison to other studies

To date, only three other studies have evaluated the cost-effectiveness of plant sterols [14, 16, 17]. A cost–benefit analysis of plant sterol-enriched low-fat margarine for cholesterol reduction based on the German population found that the 10-year CVD risk and associated costs were significantly lower for the plant sterol group compared with the normal diet group. A projection at the level of the German population led to a reduction of 117,000 CVD cases over 10 years for the whole German population and a cost saving of €1.3 billion [14]. Similar results were demonstrated in Canada, where it was estimated that significant savings could be made annually for the publicly funded healthcare system if plant sterol-enriched food was approved for sale [17]. It has also been suggested that plant sterol-enriched spreads are potentially cost-effective in the prevention of CVD risks in adult men and in older women in Finland [16].

### Limitations of the study

The results should be interpreted in light of the limitations of the study and the assumptions that were made. HSE only covers households in England so findings may not be more widely generalizable. Compliance levels were based on studies from Canada, but these may not reflect the consumption level of plant sterols in England. Complicating matters further, the duration of the effects of plant sterols on reduction in LDL-c level is not clear. Whilst some studies found that the cholesterol-lowering effect is established within a few weeks, and is proved to remain stable for at least a year [7, 8, 10], we have assumed a more enduring benefit. Research also shows that doses higher than 3 g/day could lead to negative side effects [8, 38, 39], but evidence on this is limited, and no effect has been allowed for. Furthermore, this study does not take account of potentially large inter-individual variability in absorption and turnover of non-cholesterol sterols that are increasingly under investigation. When these effects are better understood, they may influence screening policies and hence cost-effectiveness [40]. The study primarily adopted the perspective of the NHS. Societal effects associated with CVD are not included (productivity loss, family costs, social care). Consideration of these issues, however, would have increased the savings to the NHS and made sterol-enriched diets more likely to be cost-effective. The data for risk calculations used in this study are mainly derived from HSE. However, it was not possible to take account of other lifestyle factors that might affect future risks and outcomes (e.g., exercise, health awareness, smoking, alcohol consumption). Similarly, it is possible that some subjects in the survey already consume sterols in some form or another, and since the extent of this was unknown, it could not be incorporated into the analysis. Also, the time horizon was set as 20 years, while QRISK2 is based on the estimation of 10-year risk.

### The policy implications of the study

Pending results from randomized controlled trials, NICE does not recommend routine use of plant sterols and stanols for CVD prevention if the patient has already received treatment [25], but focuses instead on promoting a more natural cardio-protective diet (low fat and sugar, whole grains, fruit, vegetables, oily fish, nuts, seeds, legumes) and use of statins [41]. This position is supported by some scholars who have pointed out that plant sterols have not been shown to reduce clinical end points and suggest that prescription drugs should be preferred to stanol/sterol esters for lowering cholesterol except in borderline hypercholesterolemia [42]. Despite this, an increasing number of experts and health organizations recommend consuming plant sterols to reduce CVD risks, including the American and British Heart Associations [31, 43]. Moreover, the European Commission has acknowledged the value of sterol-enriched foods for cholesterol lowering through approval of health claims on some products [4, 6].

The findings from this study add weight to calls for the increased use of plant sterol-enriched functional food as a preventive strategy for people with hypercholesterolemia, and suggest that encouraging the consumption of plant sterol-enriched functional food is likely to bring cost savings to health systems, as well as improving patient outcomes. In England, the annual cost of a subsidy equivalent to the excess cost of sterol-enriched spread (about £57 per subject in this study) is similar to the annual cost of many statins. Although subject to debate, a drawback that is raised regarding statins is that compliance is reduced because of side effects, and that adverse events may occur [44–47]. Such issues are less likely to be relevant to functional foods. Unlike statins, however, which are provided with doctor endorsement and on prescription, efforts may be needed to ensure that consumers are aware and motivated to use sterol-enriched products and are able to understand the claims made on them. Under some circumstances, mass media campaigns may be effective, but consideration needs to be given to costs and likely impact in policy deliberations [48].

## Appendix 1

See Table 5.

**Table 5:** Cost- and health-utility values for health states

Health states	Costs (£)	Source	Utility	Source
EF	0	Ara et al. [36]	1.060–0.004*age	Ara et al. [36]
Angina first year	684.00	NHS reference costs 2013/14	0.808	Lenzen et al. [49], Ara et al. [36]
Angina subsequent years	233.00	Ara et al. [38] (inflated to 2015)	0.9	
Unstable angina first year	1428.50	NHS reference costs 2013/14	0.731	Goodacre et al. [35], Kim et al. [50]
Unstable angina subsequent years	233.00	Ara et al. [38] (inflated to 2015)	0.8	
MI first year	1377.00	NHS reference costs 2013/14	0.7	Goodacre et al. [35], Lacey and Walters [51]
MI subsequent years	233.00	Clarke et al. [57], 155 (inflated to 2015)	0.8	
TIA	1419.00	NHS reference costs 2013/14	1.060–0.004*age	Aprile et al. [52], Ara et al. [36]
TIA subsequent years	373.00	Ara et al. [38] (inflated to 2015)	1.060–0.004*age	
Stroke	4843.00	NHS reference costs 2013/14	0.5	Tengs and Lin [53], van Exel et al. [54], Leeds et al. [55]
Stroke subsequent years	3055.00	Youman et al. [56] (inflated to 2015)	0.629	

## Appendix 2

See Table 6.

**Table 6:** Calculation of the food costs

Functional food	Unit cost (£)	Plant sterols	Annual cost (£)
Plant sterol yoghurt drink 6 × 67.5 G (manufacturer’s brand)	3.78	2 g per bottle	460.2
Plant sterol yoghurt mini drink 6 × 100 ml (manufacturer’s brand)	3.5	2 g per bottle	426.13
Plant sterol Milk 1L (manufacturer’s brand)	1.39	3 g per 1 L mil2l	507.7
Plant sterol Light Spread 250 G (manufacturer’s brand)	1.9	18.75 g per 250 G spread	111.04
Non-sterol-enriched spread 500 G (supermarket’s own brand)	1.85	N/A	54.1

The calculation of annual cost is based on 3 g/day intake and 365.25 days per year. One unit (bottle) of plant sterol yoghurt drink contains 2 g plant sterols, so the daily cost is based on a consumption of 2 units. There are currently two plant sterol enriched spreads available in the UK, and costs were based on the leading brand

## Appendix 3

See Table 7.

**Table 7:** Parameters and distributions

	Base-line value	Distribution	Source
Annual CVD risks	Derived from HSE 2011 using QRISK2 function	Log-normal	Authors’ own
RR	Age- and gender-specific RR	Log-normal	Authors’ own
Transition probabilities	Age- and gender-specific transition probabilities	Beta	Derived from Ward et al. [21]
Costs of health states	Cost for the first year and subsequent year of each health state was allowed	Gamma	First-year costs were derived from NHS reference costs. Subsequent year costs were derived from Ward et al. [21]
Costs of the functional food	Supermarket price in April, 2014	Deterministic	Derived from supermarket websites in April, 2014
Utility of health states	Utilities of the first year and subsequent year of each health state was used in the analysis	Beta	Derived from Ara et al., Ward et al. [21], D’Agostino et al. [34], Goodacre et al. [35], Lensen et al., Kim et al., Lacey et al.
Compliance level	Varying compliance level at 50 and 100% for the main analysis.	Beta	Derived from National Diet and Nutrition Survey
Discount rate	3.5% for both cost and utility	Deterministic	Derived from NICE technology appraisals methods guide 2013

Parameter distributions are consistent with Briggs et al. 2014

## Appendix 4

See Table 8.

**Table 8:** ICER (£/QALY): results of sensitivity analyses

	Mild-cholesterol population		High-cholesterol population	
	Male	Female	Male	Female
NHS pays the cost difference between sterol-enriched and normal spread 50% compliance level				
LDL reduction at 10.7%				
45–54	75,009.93	251,029.38	47,023.289	149,367.506
55–64	41,170.06	117,946.26	24,539.187	77,118.267
65–74	21,675.50	37,354.67	14,832.649	25,045.405
75–84	18,506.26	25,913.87	13,069.377	18,724.213
LDL reduction at 13.3%				
45–54	64,061.41	200,643.05	40,957.433	123,877.587
55–64	34,767.54	94,212.97	21,297.750	62,882.757
65–74	18,096.92	29,982.24	12,547.142	20,458.145
75–84	15,297.62	20,403.12	11,015.281	15,031.192
NHS pays the cost difference between sterol-enriched and normal spread 50% compliance level				
Time horizon as 10 years				
45–54	144,664.38	481,921.45	98,721.807	304,097.157
55–64	73,227.38	231,149.10	46,967.747	158,458.671
65–74	36,043.75	70,154.82	25,600.421	48,643.878
75–84	19,820.82	30,012.02	13,320.681	20,488.245
Individual pays the full cost of the plant sterol-enriched spread				
5% compliance level				
45–54	− 2032.06	− 2311.43	− 2032.08	− 2311.44
55–64	− 1892.32	− 2219.71	− 1892.37	− 2219.72
65–74	− 1593.21	− 1486.11	− 1593.25	− 1486.13
75–84	− 2002.72	− 1573.18	− 2002.83	− 1573.21
NHS pays the cost difference between sterol enriched and normal spread 50% compliance level				
Time horizon as 10 year				
45–54	144,664.38	481,921.45	98,721.807	304,097.157
55–64	73,227.38	231,149.10	46,967.747	158,458.671
65–74	36,043.75	70,154.82	25,600.421	48,643.878
75–84	19,820.82	30,012.02	13,320.681	20,488.245

Plant sterol-enriched spread cost-effective at NICE threshold below £30,000 per QALY gained

Plant sterol-enriched spread cost-effective at NICE threshold below £20,000 per QALY gained
